# Changes in *Cis*-regulatory Elements during Morphological Evolution

**DOI:** 10.3390/biology1030557

**Published:** 2012-10-25

**Authors:** Stephen J. Gaunt, Yu-Lee Paul

**Affiliations:** 1 The Babraham Institute, Babraham, Cambridge, CB22 3AT, UK; Email: yulee.paul@babraham.ac.uk; 2 Laboratory for Development and Evolution, Department of Zoology, University of Cambridge, Cambridge, CB2 3EJ, UK

**Keywords:** morphological evolution, gene expression, gene regulation, *cis*-regulatory elements, CREs, enhancers

## Abstract

How have animals evolved new body designs (morphological evolution)? This requires explanations both for simple morphological changes, such as differences in pigmentation and hair patterns between different *Drosophila* populations and species, and also for more complex changes, such as differences in the forelimbs of mice and bats, and the necks of amphibians and reptiles. The genetic changes and pathways involved in these evolutionary steps require identification. Many, though not all, of these events occur by changes in *cis*-regulatory (enhancer) elements within developmental genes. Enhancers are modular, each affecting expression in only one or a few tissues. Therefore it is possible to add, remove or alter an enhancer without producing changes in multiple tissues, and thereby avoid widespread (pleiotropic) deleterious effects. Ideally, for a given step in morphological evolution it is necessary to identify (i) the change in phenotype, (ii) the changes in gene expression, (iii) the DNA region, enhancer or otherwise, affected, (iv) the mutation involved, (v) the nature of the transcription or other factors that bind to this site. In practice these data are incomplete for most of the published studies upon morphological evolution. Here, the investigations are categorized according to how far these analyses have proceeded.

## 1. Introduction

Changes in the shape or pigmentation patterns of the body provide the most dramatic examples of morphological evolution, and these commonly occur by mutations in developmental genes. The body plan of the adult is preceded by a pre-plan of developmental gene expression in the embryo. These genes are expressed in precise patterns in time and space, and it is these that instruct the cells on how they must develop. Developmental genes code, primarily, for transcription factors and signalling components, and each of these is typically deployed at multiple tissues and times in order to specify a wide range of different developmental events. Developmental genes therefore exert pleiotropic (multiple and seemingly unrelated) effects during embryonic development [1].

Temporo-spatial variety in the expression of a developmental gene is typically mediated by variety in the range of tissue-specific *cis*-regulatory elements (CREs) located around the coding sequence. These include enhancers and silencers of gene expression. Regulation is therefore modular, and mutational changes within any individual CRE can affect expression in one, or a subset of, tissue(s) without affecting expression at others. This may allow evolutionary change in the morphology of part of an animal without producing multiple (pleiotropic), and likely deleterious, effects elsewhere in the body [2,3,4]. 

The purpose of this article is to review and categorize examples of how mutation within CREs may cause morphological evolution. It is important to stress, however, that this is not the only way in which morphological evolution can occur. In particular, changes in the coding sequence of, for example, *Ubx* and *Antp* Hox genes have modified the target specificity of their encoded transcription factor proteins. This has mediated evolutionary changes in the arrangement of arthropod legs [5,6,7]. Thus, the Hox proteins are themselves modular, with an alteration in protein sequence affecting function in one tissue but not others [8]. The argument that mutations in CREs are less likely to produce pleiotropic effects than mutations in coding sequence [2,3,4], while likely valid, should not overshadow the significance of these alternative routes to the avoidance of adverse pleiotropic effects [9,10,11]. It seems likely that evolution will exploit a multiplicity of different mechanisms in order to effect morphological change [12].

In attempting to identify the mutational events that underlie a phenotypic change, there is great benefit in choosing a species where transgenic animals can be produced, permitting analysis of the effects of enhancer mutations upon both reporter gene expression and phenotypic rescue. The importance of these techniques is apparent in the examples described below.

## 2. Examples of Changes in *Cis*-regulatory Elements during Morphological Evolution

Here, we categorize the investigations according to how far they have proceeded to relate phenotypic change, CRE identification, CRE mutations, and the resultant gain or loss of functional transcription factor binding sites. The model systems described may in future change their categories as additional data become available. 

### 2.1. Evolutionary Change Recognized as Change in a *Cis*-regulatory Element of a Developmental Gene

#### 2.1.1. Stickleback Pelvic Fin Loss

**Figure 1:**
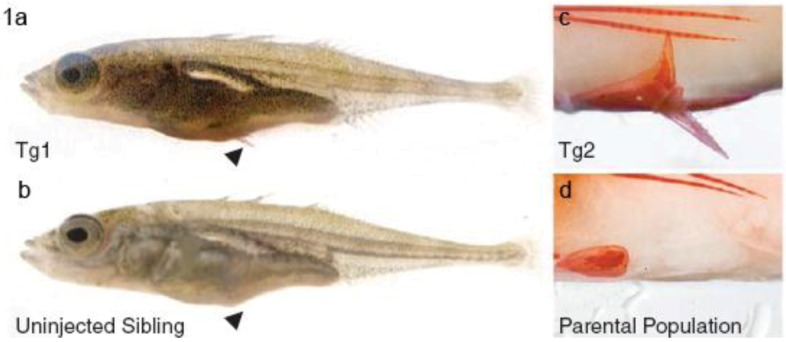
Stickleback populations have evolved loss of pelvic fins by loss of a *Pitx1* pelvis *cis*-regulatory element. Pelvic fin-negative populations (**b,d**) may be restored to an ancestral-like condition (**a,c**) (Tg1, Tg2) by expression of a *Pitx1* transgene that contains 2.5 kilobases of 5′ flanking region taken from a pelvic fin-positive population linked to *Pitx1* coding sequence from a pelvic-reduced population. Arrows in a and b show pelvic fin position. In c and d the pelvic apparatus is shown stained with alizarin red. Reprinted with permission from [13], Copyright 2010, AAAS, and D. Kingsley.

Threespine sticklebacks usually possess a robust pelvic apparatus with attached pelvic spine. However, among those living in freshwater, there has been repeated evolution of pelvic-reduced populations, which lack pelvic spines, and which may thereby become less susceptible to low calcium and to predatory arthropods which grasp the spines (Figure 1) [13]. Chan *et al*. noted that these fish have pelvis-specific depletion in their expression of *Pitx1* [13], a gene expressed in hindlimbs but not forelimbs of many different vertebrates. These authors found that an upstream pelvis CRE is commonly deleted within the *Pitx1* gene of pelvic-reduced fish populations. They showed that a 2.5 kilobase DNA fragment including this region from normal fish could, when incorporated in a *Pitx1* transgene, be used to rescue normal pelvic structures in pelvic-reduced fish (Figure 1). It appeared that the *Pitx1* enhancer may be particularly sensitive to loss due to its location in a fragile, flexible, sub-telomeric region, and that deletions in the pelvic enhancer region are subject to positive selection in multiple natural populations [13]. Change of *Pitx1* activity may also account for pelvic reduction in a mammal, the manatee [14]. 

#### 2.1.2. Forelimb Length in Bat and Mouse

Bats have forelimb skeletal elements proportionally longer than mice, and this difference develops mainly in late gestation. The ratio of forelimb length to crown rump length is similar for both animals at early stages of limb development but, by adult, becomes about 1:3 for mouse and 2:1 for bat (*Carollia perspicillata)* [15]. Cretekos *et al*. noted that the *Prx1* gene, expressed in the embryonic forelimb, promotes forelimb bone elongation, and *Prx1*-null homozygous mice have forelimbs 12.5% shorter than controls at 18.5 days [15]. In particular, these authors found that mice in which an upstream 1 kilobase CRE region of the *Prx1* gene is replaced with the orthologous bat sequence have forelimbs that are about 6% lengthened at late gestation, suggesting that mutational changes in the CRE may have been important in the evolution of the bat wing.

#### 2.1.3. Vertebral Formulae of Mouse and Chicken

Mouse and chicken differ in their vertebral formulae (relative numbers of cervical, thoracic, lumbar and sacral vertebrae). In particular, the chicken has more neck vertebrae and fewer thoracic than mouse. This is due to evolutionary shifts (transpositions) of Hox gene expression boundaries along the axial series of somites [16,17]. Boundaries within neural tissue are correspondingly transposed [18]. *Hoxc8* and *Hoxa7* are two genes whose endogenous expression boundaries in neural and somitic tissues are shifted posteriorly in chicken relative to mouse.

Belting *et al*. compared mouse and chicken *Hoxc8* CREs in their ability to direct expression of a *lacZ* reporter gene in transgenic mice [19]. They showed that the mouse 5′-located ‘early enhancer’ CRE directs anterior boundaries of lacZ expression in somitic and neural tissues at positions about four segments more anterior than those directed by the orthologous chicken CRE. These authors concluded that evolutionary transposition of *Hoxc8* expression is due to changes in its CRE. The *Hoxc8* enhancers from a variety of fish and other vertebrates display a range of mutational changes within putative transcription factor binding sites [19,20,21,22]. Some, though not all, of these provide novel lacZ expression boundaries when tested in the transgenic mouse reporter assay. These additional studies show further that there have been mutational changes within a *Hoxc8* CRE during vertebrate evolution, and that these are associated with functional changes in the *lacZ* reporter assay within transgenic mice.

It might be expected that these findings are reproducible for other Hox genes that show transpositions in their expressions in mouse relative to chicken. However, mouse and chicken *Hoxa7* CREs do not differ significantly in the anterior boundaries of neural lacZ expression that they generate in the transgenic mouse reporter assay [18]. This suggests that changes in CREs are unlikely to have been responsible for this difference in *Hoxa7* expression in mouse versus chicken. Instead, change in trans-acting factors seems more likely. 

#### 2.1.4. Loss of Vibrissae and Penile Spines in Human

McLean *et al*. used database analysis to identify more than 500 regions of high sequence conservation deleted in humans (hCONDELs) but present in chimp and macaque [23]. One of these flanks the androgen receptor (AR) locus. This region includes a highly conserved CRE which, taken from chimp or mouse, directs lacZ expression in transgenic mouse embryos to two androgen responsive sites: facial vibrissae and the genital tubercle [23]. Mice with a mutation in the AR coding sequence do not have penile spines, structures present in many mammals and thought to increase tactile stimulation. Castrated primates lose their penile spines and castrated mice have shortened facial vibrissae. Both effects are reversed by testosterone administration. Humans do not have sensory vibrissae or penile spines, and this is likely due to evolutionary loss of the AR vibrissae and penile spine enhancer [23]. Strictly speaking, verification requires rescue of this phenotype in human, as performed for pelvic spines in fish, though ethics preclude this.

### 2.2. Evolutionary Change Correlated with Mutations Located within a *Cis*-regulatory Element of a Developmental Gene

Here, the genetic basis of the morphological change has been narrowed down to mutations identified within a CRE. However, the way in which these mutations exert their effect upon morphology, for example which transcription factor binding sites they affect, is not yet clear.

#### 2.2.1. Pigmentation Differences between *Drosophila melanogaster* Populations

**Figure 2:**
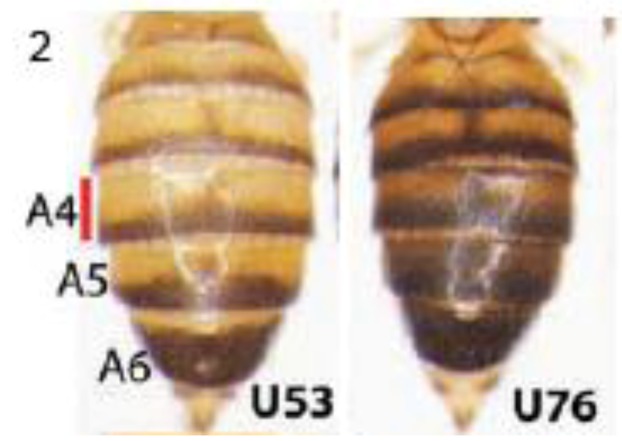
Pigmentation in different Ugandan populations of *Drosophila melanogaster* varies due to mutations in the *ebony cis*-regulatoryelement. Posterior parts of each abdominal segment are dark due to absence of *ebony* expression. Segments in the U53 population have higher overall levels of *ebony* mRNA than in U76 (names indicate the per-cent darkness of the A4 abdominal segment). At least five mutations scattered within a 5′-located regulatory element of *ebony* decrease *ebony* expression in the dark population. Reprinted with permission from [24], Copyright 2009, AAAS.

Expression of the *ebony* gene generates yellow shade in *Drosophila* adult cuticle, and its absence in posterior parts of each segment causes a dark, melanic appearance (Figure 2). Rebeiz *et al*. studied African *Drosophila melanogaster* populations that vary in the relative widths of yellow and dark bands, where increasing darkness is a derived adaptation to high altitude [24]. They examined *GFP* reporter transgenes driven by a 5′-located *ebony* CRE to show that at least five mutations affect *ebony* expression in the Ugandan populations. These include both new mutations and standing genetic variations. Four of these are single base-pair changes. They suggest that new mutations combine to create an allele of large effect, and that this may be a general feature of CRE evolution, consistent with other evidence that mutations at multiple sites within CREs are responsible for evolutionary changes in gene expression [25,26,27,28]. This may readily be explained, since CREs typically contain multiple transcription factor binding sites, distributed across a few hundred base pairs or more, and each contributes to transcriptional control. 

#### 2.2.2. Abdominal Pigmentation Differences between *Drosophila yakuba* and *D. santomea* Sister Species

**Figure 3:**
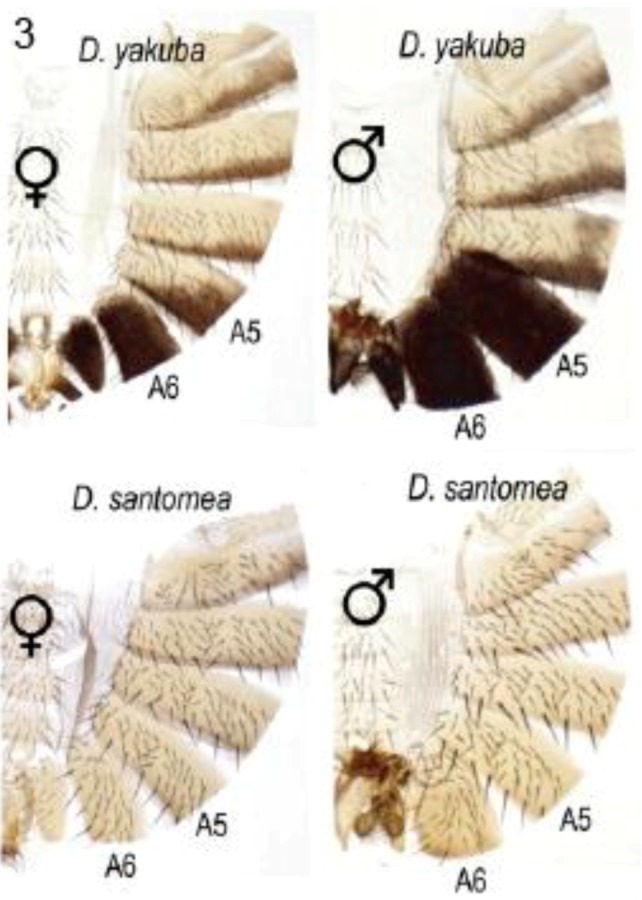
Male-specific pigmentation in *Drosophila santomea* lost in part by mutations in the *tan* gene *cis-*regulatory element. *D. santomea* and *D. yakuba* occupy the same island but only *D. yakuba* retains their shared ancestral condition of intense pigmentation in male abdominal segments A5 and A6. Abdominal cuticles are displayed with the dorsal tergites to the right. Reprinted with permission from [30], Copyright 2008, Elsevier.

In many species of the *melanogaster* group, and under control of the Abd-B Hox protein, the posterior abdominal segments of the male (A5 and A6) are strongly pigmented while those in the female are not [26,29]. *D. santomea* has lost this pigmentation, unlike *D. yakuba* which occupies lower elevations on the same island habitat [30] (Figure 3). Jeong *et al.* showed that pigmentation in *D. santomea* can be partially restored by expression from a *D. yakuba tan* transgene [30]. Two single base mutations in the 5′-located *tan* CRE were found responsible for partial loss of pigmentation in *D. santomea*. The remainder of the loss was attributed to reduction in *yellow* expression due to changes at other loci that act in *trans.* The mutant allele in the *tan* CRE was found to be one of three mutant alleles that have arisen independently in *D. santomea* populations. 

#### 2.2.3. Trichome Differences in Larvae of Different *Drosophila* Species

**Figure 4:**
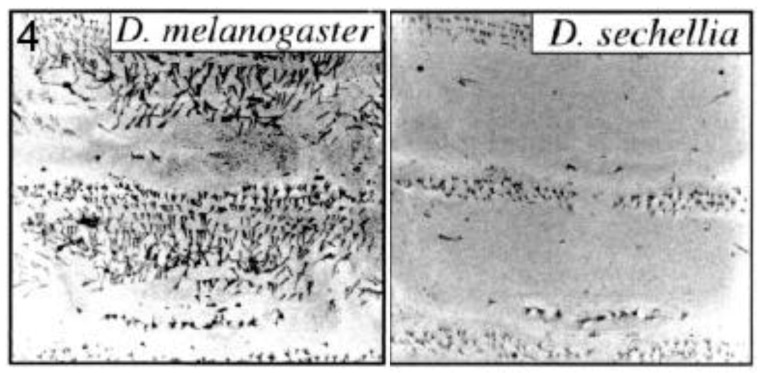
Trichome pattern on larvae of *Drosophila sechellia* lost by mutations in the *shavenbaby (svb)* gene *cis*-regulatory element. Confocal micrographs are shown for abdominal segments 1 and 2 of each species. Reprinted with permission from [31], Copyright 2000, National Academy of Sciences, USA, and D. Stern.

Small ‘hairs’ (microtrichia or trichomes) decorate the dorsal larval surface of most species of the *D. melanogaster* subgroup, but not *D. sechellia* (Figure 4). The *shavenbaby (svb)* encoded transcription factor specifies these dorsal trichomes [31]. *svb* is not expressed dorsally in *D. sechellia* due to mutations in the CRE of the *svb* gene [25]. Frankel *et al*. identified thirteen substitutions and one deletion in a 500 base pair region of high sequence conservation located within a 5′-positioned CRE of *svb* [28]. They performed transgene-mediated rescue experiments to show that at least five of the substitutions contributed to trichome development. Mutations at the sites were interactive and not simply additive in their effects. The authors propose, as already mentioned in section 2.2.1, that where the function of a CRE relies on multiple transcription factor binding sites, each with a small effect on expression, evolution may require changes of a large number of such sites to cause a significant phenotypic change. They also suggest that stepwise mutations, as opposed to large scale deletion/addition of an enhancer, may help avoid pleiotropic effects since each individual CRE may mediate expression in more than one spatial domain. 

### 2.3. Evolutionary Change Understood as Acquisition, Loss, or Changed Efficiency of Defined Functional Motifs within *Cis*-regulatory Elements

#### 2.3.1. Male-specific Wing Spots in *Drosophila biarmipes*

**Figure 5:**
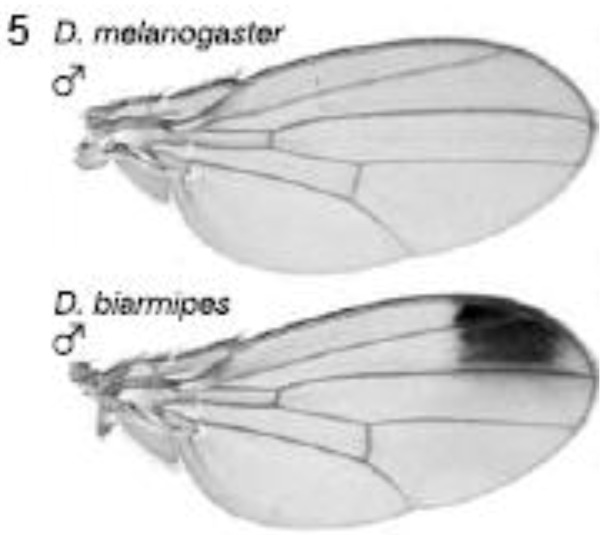
Evolution of male-specific wing spot in *Drosophila biarmipes* involved acquisition of new *Engrailed* binding motifs in the *yellow* gene *cis*-regulatory element which limit expression to the anterior compartment of the wing. Reprinted with permission from [32], Copyright 2005, Macmillan Publishers Ltd.

The *yellow* gene, required for production of black pigment, is expressed uniformly at low levels throughout the *D. melanogaster* wing, but is also expressed intensely to produce a distal anterior wing spot in *D. biarmipes,* a closely related species (Figure 5). Gompel *et al*. showed that an upstream region, the so-called ‘wing element’, regulates *yellow* gene expression [32]. These authors found that *GFP* reporter transgenes driven by the *D. biarmipes* wing element are expressed in *D. melanogaster* with a pattern rather similar to that of the wing spot in *D. biarmipes*. They showed that separate sequences in the wing element of *D. biarmipes* regulate (i) general wing expression, (ii) wing spot. Deletion and mutation studies upon the spot element showed that it contained an activator element (which promotes expression in both anterior and posterior parts of the wing) and repressor elements (which exclude the spot from the posterior wing compartment). These repressor elements, present in *D. biarmipes* but not *D. melanogaster*, have gained binding sites for the transcription factor *Engrailed,* which confine the spot to the anterior compartment. Therefore *Engrailed* has been co-opted to pattern the location of the wing spot. Gompel *et al*. suggest that, as a general mechanism for generating wing patterns, random mutation of ancestral CREs (including point and insertional mutations) generate transcription factor binding sites to modify wing pigment patterns by co-opting from the many transcription factors already present in spatially distinct patterns within the wing [32]. This is proposed not only as a mechanism for wing patterns but more importantly as a general mechanism to evolve novel patterns of gene expression for diverse traits in diverse organisms [32].

#### 2.3.2. Male-specific Abdominal Pigmentation Differences between *Drosophila melanogaster* and *D. willistoni*

**Figure 6:**
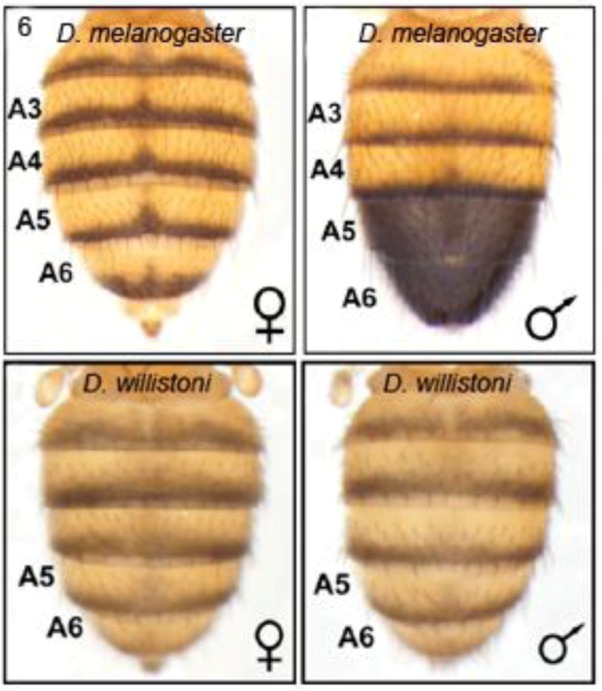
Male-specific pigmentation in *Drosophila melanogaster* and *D. willistoni* differ by ‘molecular remodelling’ of Abd-B and dsx binding motifs within the intron *cis*-regulatory element of the *bric à brac* gene, *bab1*. Reprinted with permission from [26], Copyright 2008, Elsevier.

As already indicated (section 2.2.2), male abdominal-specific pigmentation is under the control of the *Abd-B* gene. Abd-B protein directly activates *yellow* to promote melanin formation and pigmentation in abdominal segments A5 and A6 of males.In females, *bric à brac (bab)*, represses this pigmentation. The *bab1* gene is regulated by Abd-B and sexually dimorphic doublesex (dsx) proteins which bind to a *bab1* intron CRE [29]. The *Abd-B/bab/dsx* pathway forms a sexually dimorphic switch which was already present in the common ancestor of *D. melanogaster and D. willistoni*. However, the switch was subsequently utilized to regulate male specific abdominal pigmentation in the *D. melanogaster* lineage [26]. Williams *et al*. showed that *D. willistoni* and *D. melanogaster* differ in their male-specific pattern (Figure 6) due to changes in the effects of Abd-B and dsx proteins upon the *bab1* intron CRE [26]. They examined a variety of *GFP* reporter transgenes driven by the *bab1* intron CRE to show that this is not simply due to change in the number of binding motifs, but also to modification of their overall efficiency by change in the polarity and spacing of existing sites. They describe this as ‘molecular remodelling’ of a pre-existing Abd-B- and dsx-regulated CRE.

#### 2.3.3. Abdominal Pigmentation Differences between *Drosophila melanogaster* and *D. kikkawai* Species

As already indicated above, male abdominal-specific pigmentation is under the control of the *Abd-B* gene. Jeong *et al*. showed that Abd-B protein activates *yellow* expression and pigmentation by direct interaction with specific binding motifs within the 5′-located CRE in the *yellow* gene [29]. They found that *GFP* reporter transgenes driven by the *yellow* CRE are only expressed in the male-specific pattern if they include these Abd-B binding sites. In a separate mechanism from that described in sections 2.2.2 and 2.3.2, male-specific pigmentation in *D. kikkawai* has been lost by mutations which include loss of the Abd-B binding motifs within the *yellow* gene CRE [29].

### 2.4. Phylogenetic Modification/Appearance/Loss of a *Cis*-regulatory Element which is of Suggestive but Unproven Function

#### 2.4.1. Human Specific Mutations within a Limb *Cis*-regulatory Element

Prabhakar *et al*. showed that a rapidly evolving non-coding DNA element in human (human accelerated conserved noncoding sequence 1, *HACNS1*) functions as a transcriptional enhancer in mouse embryos, mediating strong expression of a *lacZ* reporter in the limbuds [27]. Expression is strong in the distal forelimb. In contrast, orthologous DNA elements from chimp and rhesus monkey drive only weak expression in the proximal limb. The authors propose that gain of function in *HACNS1* may have influenced the evolution of human limb features, such as hand dexterity and bipedalism, by altering the expression of nearby genes during limb development. Thirteen base substitutions over an 81 base region accounted for the functional difference between chimp and human elements. More recently, it has been suggested that the changes in human *HACNS1* have disrupted a repressor function rather than activated a new enhancer [33].

#### 2.4.2. Sox Response Elements in *Fezf2*, a Determinant of Corticospinal Neuron Identity

*Fezf2* encodes a zinc-finger transcription factor required for molecular specification within layers 5 and 6 of the mammalian neocortex and its corticospinal output. Shim *et al*. showed that *Fezf2* expression is driven by a CRE, named E4 [34]. Mice deleted for E4 showed down-regulation of *Fezf2* expression in the neocortex and anatomical changes as earlier reported in *Fezf2*-deficient mice. Transfection experiments in cultured cells using luciferase reporters driven by E4 showed that it contained at least one functional Sox binding motif. This Sox binding element apparently arose within the E4 CRE at the junction between fish and tetrapods. The authors propose that this event may have contributed to the evolution of the neocortex in mammals and at least some other amniotes [34]. 

#### 2.4.3. Retinoic Acid Response Elements in Two *Cis*-regulatory Elements of the Vertebrate Homeobox Gene, *Cdx1*

*Cdx1* expression in mice utilizes both upstream and intron CREs [35,36]. Homeotic genes including *Cdx1* and several anteriorly-expressed Hox genes are directly responsive to retinoic acid (RA), and contain retinoic acid response elements (RAREs) in their CREs. RA is a morphogen in chordates, though not in pre-chordates [37]. In cell culture assays upon transfected *Cdx1* reporter constructs, Gaunt and Paul found that both intron and upstream RAREs are functional in mouse DNA, yet only the intron RARE is functional in chicken [38]. Database analysis indicated that the intron RARE first appeared at the transition from amphibians to amniotes (reptiles, birds and mammals), while the upstream enhancer RARE first appeared at the transition from marsupial to eutherian mammals (Table 1) [38]. 

There is no direct evidence linking acquisition of *Cdx1* RAREs to morphological evolution of vertebrate groups, but these authors noted that the main sites of action of *Cdx1* were also important sites of distinction between vertebrate types. *Cdx1* regulates homeotic patterning of the neck vertebrae in mice, up to and including the atlas/skull level, probably by its effect upon Hox genes [39]. Like most fish, extant amphibian species differ from amniotes in lacking a distinct neck (region between the skull and pectoral girdle). These amphibians possess only one neck vertebra (atlas) and lack the rotation-permitting atlas-axis joint. Fossil evidence indicates that the latter first arose at around the amphibian/amniote split [40,41]. Acquisition of the *Cdx1* intron RARE at around this time might therefore have contributed to evolution of the vertebrate neck, including ability to lift and rotate the head in early tetrapods [38]. *Cdx1* also regulates patterning of the ureters and Müllerian ducts (precursors to the uterus). Marsupials differ from eutherian mammals in the layout of the ureters and Müllerian ducts. Acquisition of the *Cdx1* upstream RARE at the marsupial/eutherian split may therefore have facilitated morphological evolution of the eutherian reproductive tract [38]. These proposed effects of *Cdx1* upon morphological evolution remain hypotheses, awaiting experimental verification. 

**Table 1:** Retinoic acid response elements (RAREs) in vertebrate *Cdx1* and Hox gene *cis*-regulatory elements. Black ticks indicate RAREs described in the literature. Red ticks indicate RAREs identified from our own database analysis (supplementary Figure 1). DR2 and DR5 indicate RAREs where the paired 6-base binding motifs are separated by either 2 or 5 spacer nucleotides, respectively, and these are located either upstream (5′) or downstream (3′) of the coding region. In general, most Hox RAREs are found to be conserved from fish to mammals, although we note that (i) some species have apparently lost a RARE even though it was present in their ancestry, (ii) the fish species examined do not have an obvious *Hoxa1* RARE homologue. It may be that this was lost subsequent to genome duplication in ray finned fish [42] since, as shown in zebrafish, *Hoxa1* function for development of the hindbrain has become adopted by *Hoxb1b* [43,44]. *, likely *Hoxa1a* of zebrafish [45]. This 3′ DR5 may not be homologous with that in the other vertebrates shown since it is of opposite sense. **, likely *Hoxb1a* of zebrafish [45]. B23-biology-01-00557, likely *Hoxd4a* of zebrafish [46].

	Fish	Amphibian	Lizard	Bird	Marsupial	Eutherian	References
***Cdx1* upstream**						✓	[47], [38]
***Cdx1* intron**			✓	✓	✓	✓	[38]
***Hoxa1* (3’ DR5)**	?*	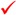	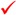	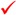		✓	[45]
***Hoxa4* (5’ DR5)**	✓	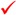	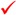	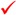	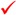	✓	[48]
***Hoxb1* (3’ DR2)**	✓			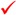	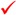	✓	[49]
***Hoxb1* (3’ DR5)**	✓^**^	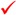		✓	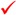	✓	[50], [45]
***Hoxb1* (5’ DR2)**				✓		✓	[51]
***Hoxb4* (3’ DR5)**	✓	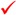	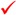		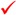	✓	[52]
***Hoxb5* (3’ DR5)**	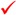		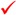		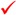	✓	[53]
***Hoxd4* (5’ DR5)**	✓^***^	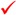	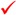	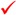	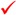	✓	[46]
***Hoxd4* (3’ DR5)**	✓^***^	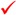	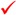	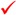	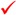	✓	[46]

#### 2.4.4. Human-specific Loss of a CRE in the *GADD45G* Tumour Suppressor Gene

McLean *et al*. described a conserved region deleted in human (hCONDEL) from a position next to the tumour suppressor gene *GADD45G* [23]. When taken from chimp or mouse, this DNA region functions as an enhancer to drive lacZ expression in subventricular zones of transgenic mouse embryo forebrain. It contains forebrain-specific p300 transcription factor binding sites. *GADD45G* normally suppresses the cell cycle. The authors propose that this deletion provides a plausible molecular basis for increased production of particular neuronal cell types in subventricular zone regions, a suggested requirement in evolutionary expansion of the neocortex in primates [23]. 

## 3. Discussion

### 3.1. Modification of *Cis*-regulatory Elements Revealed as a Mechanism Used in Morphological Evolution

Experiments reviewed above show how CREs have become modified in order to permit new patterns of gene expression and new morphologies [54]. This occurred by acquisition of new transcription factor binding sites (section 2.3.1), loss of transcription factor sites (section 2.3.2), molecular remodelling of sites (section 2.3.3), acquisition of multiple point mutations (sections 2.2.1, 2.2.2, 2.2.3), or loss of the entire region (section 2.1.1, 2.1.4). It is suggested that loss of the entire CRE, as opposed to multiple point mutations, may contribute to morphological evolution only rarely because many single CRE ‘modules’ do in fact drive expression in multiple domains [9], and their loss will therefore produce pleiotropic effects [28]. The studies identify more examples of evolutionary loss, rather than gain, of CRE function. This likely indicates that gain of function occurs more rarely [3].

Of the above studies, those on *Drosophila* in particular were facilitated by comparing closely related populations differing by relatively simple morphologies, such that significant mutations were less likely to be masked by secondary genetic changes [55]. A limitation with this approach, however, is that we often wish to compare species more distantly related and with widely divergent morphologies.

### 3.2. A Problem in Comparing *Cis*-regulatory Elements of Distantly Related Species

The studies on evolutionary transposition of *Hoxc8* and *Hoxa7* expression boundaries (section 2.1.3) reached different conclusions about the likely role of changes in CREs. The experiments were based upon comparing chicken and mouse regulatory regions as drivers of *lacZ* reporter expression in transgenic mice. The contrasting results may indeed indicate a true difference between the evolutionary histories of the two genes being studied. However, the experiments reported do not rule out problems in cross species reporter assays. 

This was recognized by Morrison *et al*. in their studies upon *Hoxb4* [56]. Endogenous *Hoxb4* is expressed in somitic mesoderm to a similar anterior boundary (the presumptive axis at the level of somite 7) in both mouse and chicken [56,57]. In somitic mesoderm of transgenic mice, a *lacZ* reporter transgene driven by a mouse *Hoxb4* CRE is expressed to this level [57], whereas a *lacZ* reporter driven by the corresponding chicken CRE has a boundary of expression that is more posterior by several segments [56]. CREs typically bind several different transcription factors and co-factors. As suggested by Morrison *et al*. it is not clear that these factors in mouse can function optimally on chicken DNA sequences since binding sites and their cognate factors may have evolved in concert within each species [56]. An effect observed in cross-species reporter assays might therefore reflect this general incompatibility rather than a specific regulatory mutation that has been important in evolution of axial morphology. This potential problem likely increases with evolutionary distance in time. Chickens and mice are separated by ~300Myr [58]; bats and mice by ~90Myr [15]. The problem with the assay of chicken CREs in mice might be alleviated if it were possible to perform the reciprocal experiment in transgenic chickens. Expression of *lacZ* reporter transgenes driven by mouse *Hoxc8* CRE should then be located anterior to that of endogenous chicken *Hoxc8*.

### 3.3. Evolutionarily Origins of *Cis*-regulatory Elements Identified in Database Searches

**Figure S1:**
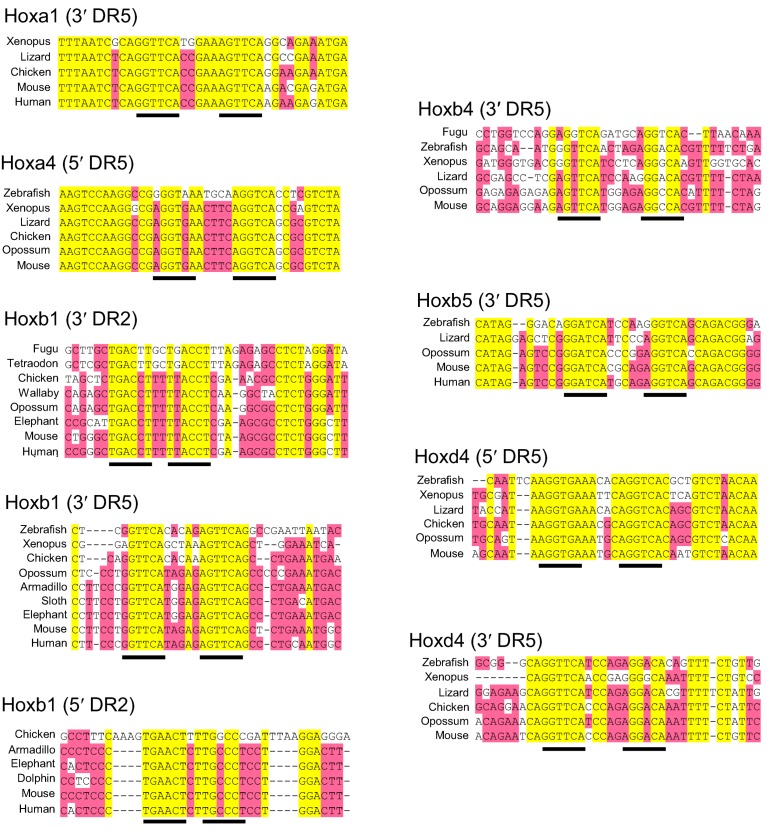
Retinoic acid response elements (RAREs) in vertebrate Hox gene enhancers. The hexamer repeats are underlined. Yellow highlight, fully conserved bases; red, highly conserved. Sequences, from ENSEMBL or NCBI databases, are aligned using Vector NTI. Lizard is Anole lizard; Armadillo is nine-banded armadillo; Sloth is two-toed sloth.

We have reviewed, in section 2.4, studies in which CREs have been identified functionally in the mouse, and then their evolutionary origins inferred from analyses upon DNA sequence databases. New species are rapidly being added to the databases, and so it seems likely that, as for *Cdx1* and *Fezf2* (sections 2.4.3 and 2.4.2), many other genes will soon be found to have acquired enhancers at points of morphological evolution, and that these will provide clues about the underlying mechanisms. Newly acquired enhancers within the Hox genes would be of obvious interest with regard to morphological evolution, and we have considered this with respect to RAREs. Like *Cdx1*, Hox genes of paralogous groups 1 to 5 contain RARE motifs and are functional in patterning of the vertebrate neck. Table 1 summarizes findings from both the literature and our own database survey (supplementary Figure 1). For most Hox genes it is clear that RARE motifs are conserved from fish to mammals and, unlike in *Cdx1*, they do not originate at intermediate phylogenetic stages. *Cdx1* may therefore be special in that its RAREs may have played roles in vertebrate morphological evolution [38]. Database studies have also been used to identify conserved DNA regions that have become deleted during human evolution [23]. Over 500 such deletions occur in non-coding regions, especially near genes involved in steroid hormone signalling and neural function. So far, two of these have been shown to be in CREs likely to be involved in morphological evolution (sections 2.1.4. and 2.4.4.) [23].

### 3.4. Problems in Elucidating the Evolutionary Basis of Widely Divergent Morphologies

For distantly-related species showing widely divergent morphologies there are substantial challenges in attempts to assess the evolutionary significance of changes in CREs. Examples discussed above include evolution of the bat wing, the human brain, the amniote neck, and the eutherian reproductive tract. It is likely that evolution proceeds through multiple small steps, or ‘successive slight modifications’ [59]. Reversal of any one of these modifying mutations may not substantially reverse the phenotype due to the multiplicity of genes involved, and because individual gene functions may be redundant due to acquisition of overlapping functions by related genes.

Replacement of the mouse *Prx1* enhancer with that of the bat does not result in a mouse with bat-like wings but it does produce a slight lengthening of the forelimbs [15]. This is a valuable indication of at least one component part in the evolution of the bat wing. With regard to *Cdx1*, mutation of the upstream RARE in mice has not been reported to have an effect upon the female reproductive tract [36]. The intron RARE has not yet been mutated in mice, but it might be surprising if this allowed ‘reverse engineering’ of a minimal, amphibian-like neck since complete loss of *Cdx1* activity does not produce this effect [60]. C*dx1* knockout mice have shown detachment of the odontoid peg from the axis [60] but it is unclear whether this relates to a similar pattern seen in fossils as a transitional state during the evolution of the amniote atlas-axis joint [41]. Overlapping gene function is a further problem in analysis of *Cdx1* function. For example, *Cdx2* plays similar roles to *Cdx1* in patterning the mouse neck, though only as far forward as the axis/atlas joint [39], and also the female urogenital system [38]. An alternative analytical approach might lie in attempting to genetically manipulate amphibians by adding gene functions known to be involved in development of the amniote neck. For example, it may be possible to induce multiple pre-thoracic vertebrae (a neck) in this way.

## 4. Conclusions

There have been many successful attempts over recent years to identify changes in CREs that underlie morphological evolution. There has been great benefit in choosing species or populations which (i) are closely related, so that mutations are less likely to be obscured by secondary genetic changes, (ii) differ by relatively simple morphologies, and (iii) are amenable to transgenesis, to permit analysis of mutation effects upon gene expression and function. In particular, comparison of *Drosophila melanogaster* populations and different *Drosophila* species has provided clear links between phenotype and genotype, and provided important illustration of the mechanistic principles of morphological evolution. Comparison of distantly related species with widely divergent morphologies is more challenging, yet the rapidly increasing number of species genomes sequenced means that CREs identified in one species can readily be traced in their phylogenetic origins. Although this can provide interesting clues about how CRE evolution may have changed phenotype, there are substantial problems in verification. Three difficulties discussed are (i) problems in cross-species enhancer assays, (ii) the multi-step nature of evolution and the multiplicity of genes that may be involved in complex traits, (iii) masking effects of overlapping gene functions. Although it is uncertain how we shall finally unravel the genetic changes involved in the transition between major animal groups, the identification of newly evolved CREs will provide us with many clues.
